# Motor unit potentials of the erector spinae muscle by concentric needle electromyography

**DOI:** 10.1002/brb3.627

**Published:** 2017-02-16

**Authors:** Andreas Posa, Izabela Niśkiewicz, Alexander Emmer, Yorck Kluge, Malte E. Kornhuber

**Affiliations:** ^1^ Department of NeurologyMartin‐Luther‐University Halle‐WittenbergHalle (Saale)Germany

**Keywords:** biceps brachii muscle, concentric needle electromyography, erector spinae muscle, motor unit potential, vastus lateralis muscle

## Abstract

**Introduction:**

The needle electromyography (EMG) serves to supply additional information in patients with suspected neuromuscular disorders. We aimed to provide motor unit potential (MUP) data by concentric needle EMG in the erector spinae (ES) in comparison with biceps brachii (BB) and lateral vastus (LV).

**Methods:**

Electromyography MUP data (*n*) were obtained in ES (517), BB (539), and LV (627) in 32 healthy volunteers (16f).

**Results:**

Motor unit potential data: amplitude (μV) 393 ± 174 (ES), 375 ± 162 (BB), and 577 ± 304 (LV); duration (ms) 10.4 ± 2.4 (ES), 10.1 ± 2.1 (BB), and 11.1 ± 2.3 (LV), area (μV × ms) 585 ± 327 (ES), 538 ± 267 (BB), and 881 ± 492 (LV); phase number 3.23 ± 0.94 (ES), 2.98 ± 0.76 (BB), and 3.19 ± 0.81 (LV); size index 0.60 ± 0.56 (ES), 0.51 ± 0.53 (BB), and 0.96 ± 0.55 (LV). LV displayed higher values (*p* at least <.001) for MUP amplitude, duration, area, and size index as compared to both, BB and ES.

**Conclusion:**

Concentric needle EMG investigations in healthy adult human subjects revealed similar MUP parameters in the ES and BB muscles, while in the LV muscle MUP amplitude, duration, area, and size index were significantly larger. Different neuromuscular disorders display a predominant involvement of proximally located muscles such as truncal muscles. The present results given here may facilitate the diagnosis of neuromuscular disorders.

## 1 Introduction

The needle electromyography (EMG) technique serves to supply additional information in patients with suspected neuromuscular disorders. Most spinal muscular atrophy patients and those with different myopathy disorders display a predominant involvement of proximally located muscles such as truncal muscles or those in the shoulder and hip regions. For concentric needle electrode investigations, normative values have repeatedly been published in few muscles such as biceps brachii (BB), or lateral vastus (LV) muscles (Barkhaus, Periquet, & Nandedkar, 1997; Bischoff, Stålberg, Falck, & Eeg‐Olofsson, 1994; Doherty & Stashuk, 2003; Finsterer & Fuglsang‐Frederiksen, 2000; McGill & Dorfman, 1985; Mische, 2014; Nandedkar, Barkhaus, & Charles, 1995; Podnar, 2009). Few EMG data are available about motor unit potentials (MUPs) in the truncal muscles (Barkhaus et al., 1997; Mische, 2014; Tomasella, Crielaard, & Wang, 2002; Travlos, Trueman, & Eisen, 1995). In fact, only one study has previously been done with concentric needle electrodes in 11 healthy subjects (Barkhaus et al., 1997). In this study, we aimed to close this gap.

## 2 Methods

Inclusion criteria were an age between 18 and 60 years, a healthy state with no hints for cognitive disturbances, neuromuscular disorders, diabetes mellitus, hemorrhagic diatheses , no medication intake, and normal findings on a neurological examination. Myopathies with axial predominance and also adult forms of spinal muscular atrophy frequently become manifest in this age range. Concentric EMG needle electrodes (50 mm × 26 Gauge disposable concentric needle electrode, CareFusion, Teca™ elite; recording area 0.07 mm²) were used throughout (Neuroscreen™, Viasys Healthcare, Höchberg, Germany). Filter settings were 5 Hz–5 kHz at a time resolution of 10 ms per division (sample rate 10 kHz). Signals were amplified with 100 μV per division or with 200 μV per division where necessary. All data were obtained and evaluated by an experienced expert in electromyography (MEK). In all subjects, the BB muscle, the LV muscle, and the erector spinae muscle (ES) were recorded prospectively. The presence of spontaneous activity was checked for in each recorded muscle. Subjects were asked to moderately activate the recorded muscle in order to record 3–5 MUP simultaneously at each recording site. It was intended to record 15 such MUP from each recorded muscle. Recordings were stored.

In a second step, MUP was isolated based on a template matching and decomposition algorithm similar to the previous report(Bischoff et al., 1994). All single MUP potentials that contributed to an averaged MUP were visualized. MUP with contaminating separate MUP(s) were eliminated by mouse click in order to get average MUPs that clearly reflected the corresponding “true” MUPs. Left‐sided BB, LV, and ES were recorded in all 32 volunteers (16 females) at room temperature (23°C). Subjects were in the supine position for the BB and LV. For the ES, subjects were positioned on the right side with the back maximally bent by flexing the hip and the head. The needle was inserted perpendicular to the skin surface, two fingers (3 cm) lateral to the midline between the spinous processes of the first and the second lumbar vertebra to a depth of 2.5 up to 4.5 cm. MUP was not recorded close to superficial sites of the muscle since this could have an impact on the measured parameters (Falck, Stålberg, & Bischoff, 1995).

No ultrasound device was used to verify the exact needle location as this is not usually available for routine purposes in any EMG laboratory where reference data are taken into consideration. Force was generated by isometric contraction of the back muscles by erecting the back in combination with slowly extending the hip in a way that 3–4 MUPs with rise times between 200 and 800 μs were available for recording. Rise times and slope values were recorded for each MUP. Amplitude, duration, area, and phase number were taken by setting cursors on screen. The size index was calculated according to Sonoo and Stalberg as 2 × log10 (amp) + area/amp (Sonoo & Stålberg, 1993). Statistical analyses were done using Kruskall–Wallis test with Mann–Whitney *U*‐test post hoc.

## 3 Results

Thirty‐two healthy subjects volunteered in the investigation after giving written informed consent. The study was approved by the ethics committee of the Martin Luther University of Halle‐Wittenberg. Males were 34.9 ± 10.6 years old and females were 31.9 ± 9.8 years old. In no instance was pathological spontaneous activity identified. Data of 1683 MUP were obtained in 32 volunteers (16 females), with a mean age of 33.4 ± 10.2 years (range 22–57 years). There was no statistically significant gender difference in age. In the ES muscle, 517 MUPs were recorded (16.2 ± 5.2 per subject). The respective values were 539 MUPs (16.9 ± 5.3) for the BB, and 627 MUPs (19.6 ± 5.7) for the LV.

**Figure 1:**
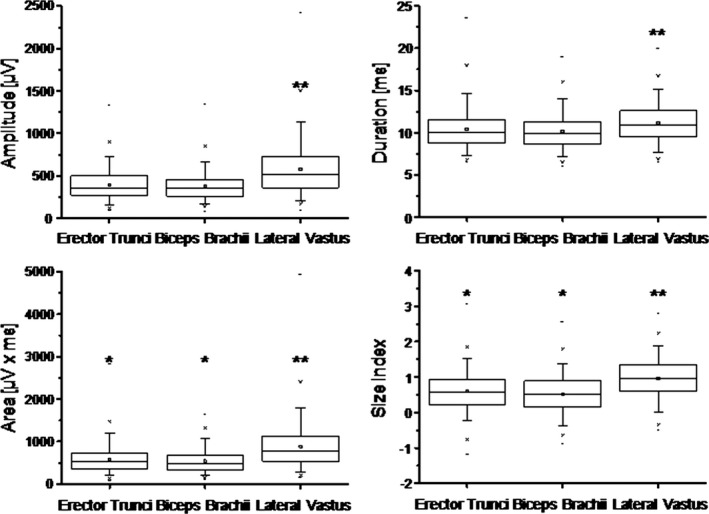
Illustration of motor unit potential data in erector spinae, biceps brachii, and lateral vastus muscles. Box, 25%–75%; whisker interval, 5%–95%; square, mean; line, median; ‐ minimum, maximum. *p < .05; **p < .001

A summary of results obtained for MUP amplitude, area, duration, phase, and size index is given in Table 1 and Figure 1. As no statistically significant differences were found for all of these parameters between males and females, data were taken and treated together. The analysis of variance (Kruskall–Wallis test) revealed statistically significant differences between the investigated muscles for amplitude, area, duration, phase, and size index. The post hoc analyses (Mann–Whitney *U*‐test) showed that results obtained for ES and BB did not significantly differ between each other for amplitude and duration while the values for area, size index, and phase number were slightly and statistically significantly larger for the ES as compared with BB (Figure 1).

**Table 1:** Mean values (standard deviation) of motor unit potential parameters in the erector spinae at the thoracolumbar level, biceps brachii, and lateral vastus compared to according values given in the literature

Author, year	Amplitude (μV)	Area (μV × ms)	Duration (ms)	Phases (*n*)	Size index
Erector spinae muscle					
Present study	393 ± 174	585 ± 327	10.4 ± 2.4	3.23 ± 0.94	0.60 ± 0.56
Travlos et al. (1995)^a^	1300 ± 1298	n.d.	5.2 ± 2.1	4.7 ± 2.2	n.d.
Barkhaus et al. (1997)					
Medial	563 ± 114	851 ± 317	9.3 ± 1.4	2.6 ± 0.3	n.d.
Lateral	462 ± 41	795 ± 76	10.8 ± 1.0	2.5 ± 0.2	n.d.
Tomasella et al. (2002)^b^	687 ± 228	n.d.	12.5 ± 1.9	n.d.	n.d.
Mische (2014)	468 ± 263	723 ± 438	10.7 ± 2.7	3.09 ± 0.83	0.78 ± 0.61
Biceps brachii muscle					
Present study	375 ± 162	538 ± 267	10.1 ± 2.1	2.98 ± 0.76	0.51 ± 0.53
Bischoff et al. (1994)	436 ± 115	n.d.	9.9 ± 1.4	2.62 ± 0.31	n.d.
Nandedkar et al. (1995)	364 ± 296	603 ± 502	10.6 ± 5.0	2.14 ± 0.99	n.d.
Barkhaus et al. (1997)	370 ± 151	622 ± 307	10.4 ± 1.1	2.1 ± 0.2	n.d.
Finsterer and Fuglsang‐Frederiksen (2000)	214 ± 54	n.d.	14.2 ± 1.8	n.d.	n.d.
Doherty and Stashuk (2003)	325 ± 84	n.d.	10.8 ± 1.5	2.5 ± 0.2	n.d.
Mische (2014)	461 ± 219	718 ± 401	10.9 ± 2.7	2.95 ± 0.67	0.72 ± 0.63
Lateral vastus muscle					
Present study	577 ± 304	881 ± 492	11.1 ± 2.3	3.19 ± 0.81	0.96 ± 0.55
Bischoff et al. (1994)	687 ± 239	n.d.	11.7 ± 1.9	3.04 ± 0.28	n.d.
Doherty and Stashuk (2003)	487 ± 137	n.d.	12.9 ± 1.7	2.7 ± 0.2	n.d.
Mische (2014)	604 ± 332	997 ± 592	11.8 ± 2.7	3.15 ± 0.73	1.10 ± 0.64

n.d., not determined.

a Values from the lumbar paraspinal muscles were obtained by unipolar EMG needle electrodes, and by filter settings of 500 Hz/10 kHz.

b Values from the lumbar paraspinal muscles (L3 segment) were obtained by unipolar EMG needle electrodes.

All MUP values measured in the LV were statistically significantly larger as compared to both, ES and BB, except for the number of phases, which did not differ significantly between LV and ES (Figure 1). When only MUP with rise times up to 600 μs were taken into consideration, mean MUP values for amplitude, duration, and area showed a small decrease for all three muscles, while values for the size index of BB and ES showed a decrease in roughly 20% and that of VL decreased by 7%. No principle differences were seen in the outcomes of statistical analyses.

## 4 Discussion

Routine EMG investigation of truncal muscles has been proposed to be mandatory in patients with, for example, inflammatory muscle disease or amyotrophic lateral sclerosis (Bromberg, 2004). The main point put forward against the routine investigation is the lack of normative values (Trojaborg, 2004). Only one study is available based on a small sample of 11 volunteers with concentric needle electrodes and with a similar template matching‐based decomposition technique as used in our study (Barkhaus et al., 1997). Two further studies have been done with unipolar electrodes (Tomasella et al., 2002; Travlos et al., 1995). The resulting data cannot be directly compared with that obtained by concentric needle electrodes due to the differences in muscle volume contributing to the recorded potentials (cf. Table 1). Therefore, we aimed to give normative values for MUP parameters with concentric needle electrodes in the ES muscle in comparison to BB and LV muscles based on a larger sample of 32 healthy subjects. The ES at the level of the first lumbar vertebra may especially refer to the longissimus dorsi muscle.

It can, however, not be completely excluded that some motor units of the multifidi muscles were also recorded since measurements were not done with sonographic control of the exact needle position. All investigations and measurements were done by a single experienced investigator (MEK) to reduce possible factors of influence. For the ES, the thoracolumbar site was chosen, since it is easily and rapidly exposed in the routine setting, and since radicular lesions are not usually expected here. For the same reason, we did not aim to study the multifidi muscles, that are known to possess a predominantly monoradicular innervation. The main finding of the present investigation was that MUP parameters of the ES muscle at the thoracolumbar region are quite similar to those obtained in the BB muscle, while MUP amplitude, duration, and area values were significantly smaller as compared to the LV (Figure 1).

Our results are in a similar range as has been reported previously, especially when standard deviations are taken into consideration (Table 1). The data obtained in this study are further corroborated by the findings of Mische (2014), who obtained similar MUP values with concentric needle electrodes in the ES and in the BB muscles while those of the LV were significantly larger in amplitude, area, and size index (Table 1). In that retrospective study, MUP data of the ES, BB, and LV muscles were obtained from a total of 184 patients who were finally diagnosed with functional disorders devoid of organic basis (Mische, 2014). A concentric needle EMG study of another truncal muscle, the rectus abdominis, based on 110 adult healthy subjects revealed MUP values that were similar to those in the ES in the present investigation, namely 374 ± 56 μV for the amplitudes and 10.0 ± 1.1 ms for the duration (Xu et al., 2007).

When a concentric needle electrode is used, the recorded muscle volume has been estimated to be restricted to a radius of about 2.5 mm in a computer simulation (Nandedkar, Sanders, Stålberg, & Andreassen, 1988). Changes in MUP amplitude and area in this situation critically depend on the density of muscle fibers per volume unit, and on muscle fiber dimensions (Stålberg & Karlsson, 2001). The diameters of both, type I und type II fibers have previously been determined in human muscles from autopsy specimens (Polgar, Johnson, Weightman, & Appleton, 1973). In the LV muscle, the diameters tended to be larger than in the ES and in the BB muscles, respectively (Polgar et al., 1973). The larger MUP amplitude and area values determined for the LV as compared to BB and ES muscles in the present investigation may be due to such differences in muscle fiber dimensions. Nevertheless, we cannot exclude that the muscle fiber density would also differ between LV and the other two muscles.

Motor unit potential parameters such as amplitude, duration, and phase number as given in Table 1 are generally used for routine purposes to discriminate states of disease from the healthy range. Other MUP values such as area or size index have been introduced as they are better suited for this purpose (Sonoo & Stålberg, 1993; Takehara, Chu, Li, & Schwartz, 2004; Zalewska & Hausmanowa‐Petrusewicz, 2000). However, they are not widely used, evtl. as they are less concrete. In conclusion, concentric needle electrode investigations in healthy adult human subjects revealed similar MUP parameters in the ES and BB muscles while MUP amplitude, duration, area, and size index were significantly larger in the LV muscle.

## Conflict of Interest

None. On behalf of all authors, the corresponding author states that there is no conflict of interest.
